# Supplementary material for: Host cell membrane proteins located near SARS-CoV-2 spike protein attachment sites are identified using proximity labeling and proteomic analysis
Source: J Biol Chem. 2022 Sep 22;298(11):102500. doi: 10.1016/j.jbc.2022.102500 (PMC9492400; doi:10.1016/j.jbc.2022.102500)
Supplement: supporting information [file mmc1.pdf]

SUPPORTING INFORMATION

Host cell membrane proteins located near SARS-CoV-2 spike protein attachment sites are identified using proximity labeling and proteomic analysis

Norihiro Kotani[1, 2*], Takanari Nakano[2], and Ryusuke Kuwahara[3]

From the [1]Medical Research Center, Saitama Medical University, 38 Morohongo, Moroyama-machi, Iruma-gun, Saitama 350-0495, [2]Department of Biochemistry, Saitama Medical University, 38 Morohongo, Moroyama-machi, Iruma-gun, Saitama 350-0495, Japan, [3]Quantum Wave Microscopy Unit, Okinawa Institute of Science and Technology Graduate University, 1919-1 Tancha, Onna-son, Kunigami-gun, Okinawa, 904-0495, Japan.
*Corresponding author email address: kotani@saitama-med.ac.jp

Fig. S1 Characterization of ACE2-expressing HEK293T cells
Fig. S2 Characterization of the single candidate molecule-expressing HEK293T cells
Fig. S3 Characterization of the ACE2- and candidate molecule-coexpressing HEK293T cells
Fig. S4 *In vitro* infection assay of SARS-CoV-2 pseudovirus for ACE2-DPP4 cells
Fig. S5 Entire images of cropped western blot data of Fig. 6A and Supporting Fig. 4

Table S1 Raw data of MS analysis for Caco-2 cells; first MS analysis
Table S2 Raw data of MS analysis for Caco-2 cells; second MS analysis
Table S3 Raw data of MS analysis for A549 cells; first MS analysis
Table S4 Raw data of MS analysis for A549 cells; second MS analysis
Table S5 Identified membrane proteins involved in SARS-CoV-2 infection
Table S6 Mass spectrometry system parameters and search parameters used in this study

A

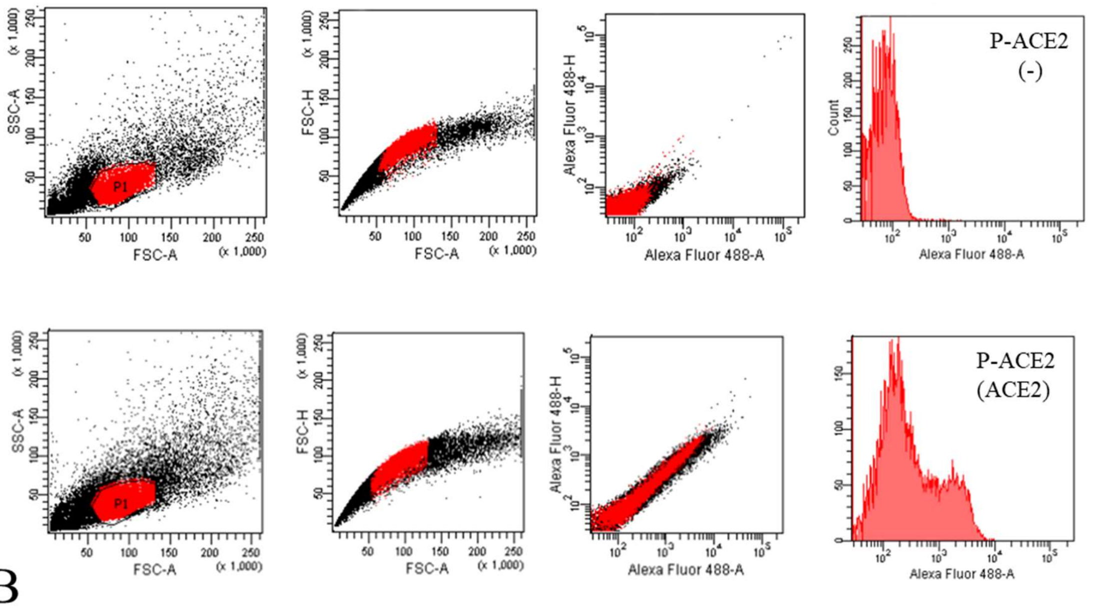
SSC-A
FSC-A
(x 1,000)
P1
FSC-H
Alexa Fluor 488-H
Alexa Fluor 488-A
Count
P-ACE2
(-)
P-ACE2
(ACE2)

B

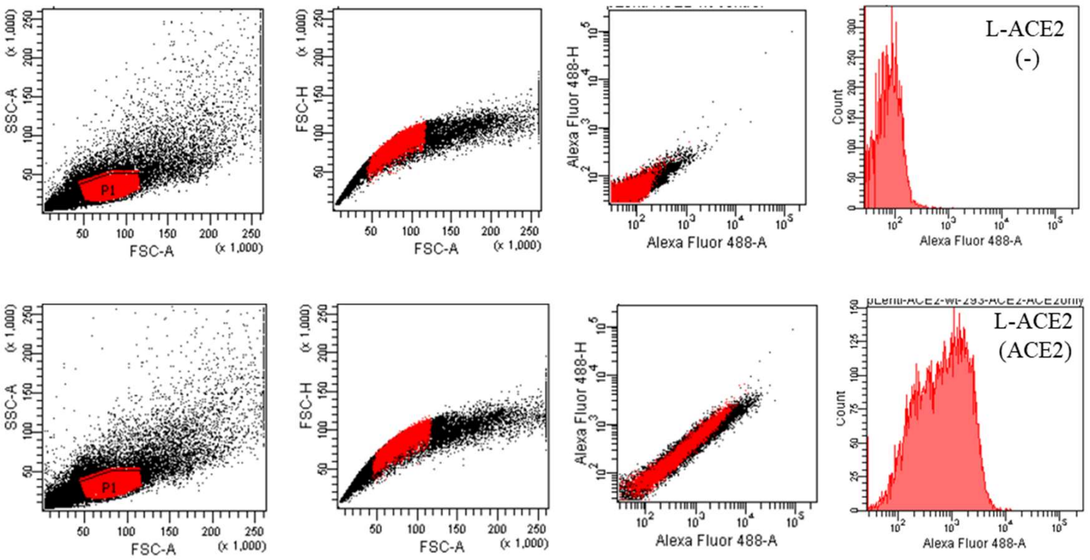
SSC-A
FSC-A
(x 1,000)
P1
FSC-H
Alexa Fluor 488-H
Alexa Fluor 488-A
Count
L-ACE2
(-)
L-ACE2
(ACE2)

C

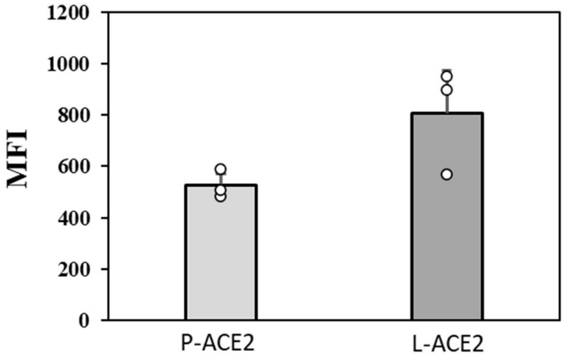
1200
1000
800
600
400
200
0
MFI
P-ACE2
L-ACE2

D

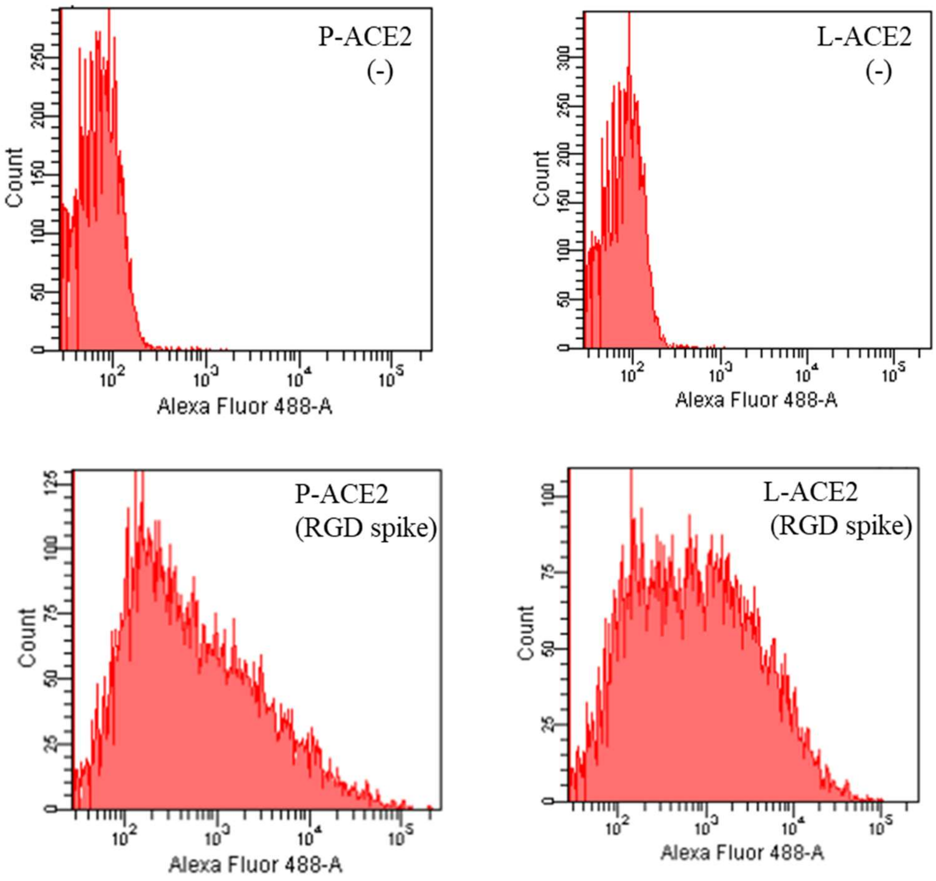
P-ACE2
(-)
L-ACE2
(-)
P-ACE2
(RGD spike)
L-ACE2
(RGD spike)
Count
Alexa Fluor 488-A

E

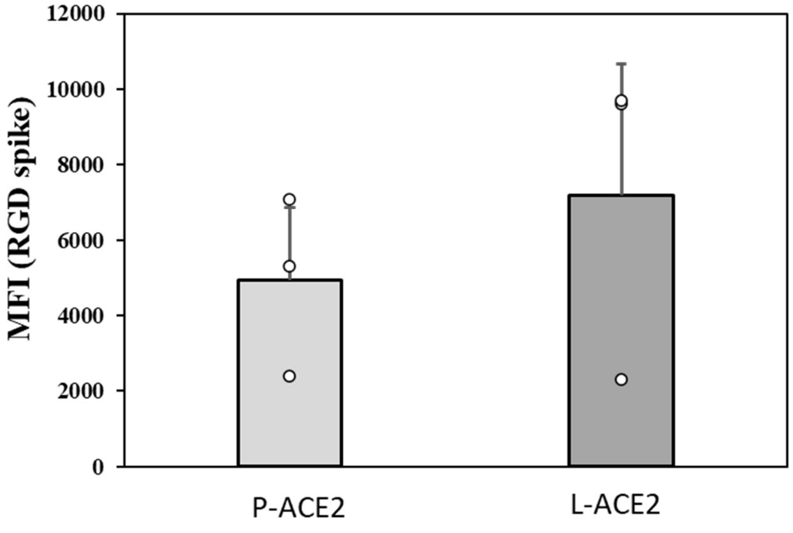
12000
10000
8000
6000
4000
2000
0
MFI (RGD spike)
P-ACE2
L-ACE2

Fig S1 Characterization of ACE2-expressing HEK293T cells

(*A, B*) Representative data from flow cytometric analysis of P-ACE2 (*A*) and L-ACE2 (*B*) cells. P-ACE2 and L-ACE2 cells were treated with an anti-ACE2 antibody, followed by incubation with Alexa Fluor 488-conjugated anti-rabbit IgG (P-ACE2 (ACE2) and L-ACE2 (ACE2); lower panel), or without anti-ACE2 antibody (P-ACE2 (-) and L-ACE2 (-); upper panel). The samples were subsequently subjected to flow cytometric analysis (Forward Scatter (FSC) vs. Side Scatter (SSC) plots, Alexa Fluor 488 plots, and Histogram of cell count vs. fluorescein intensity). Three independent experiments were performed. (*C*) Comparison of the mean fluorescence intensity (MFI) between P-and L-ACE2 cells. The MFI (ACE2) in L-ACE2 cells with anti-ACE2 antibody staining (right bar) was higher than that in P-ACE2 cells (left bar). (*D*) Representative data from flow cytometric analysis of the spike protein-treated P-ACE2 and L-ACE2 cells. The cells were treated with SARS-CoV-2 spike protein, followed by incubation with Alexa Fluor 488-conjugated mouse IgG second antibody (P-ACE2 (RGD spike) and L-ACE2 (RGD spike); lower panel), or without spike protein (P-ACE2 (-) and L-ACE2 (-); upper panel). The cells were subsequently subjected to flow cytometric analysis. Three independent experiments were performed. The data of "P-ACE2 (-)" is the same as the upper right panel of Fig S1A because of the same experimental set. (*E*) The MFI (RGD spike) in L-ACE2 cells (right bar) with spike protein staining was higher than that in P-ACE2 cells (left bar).

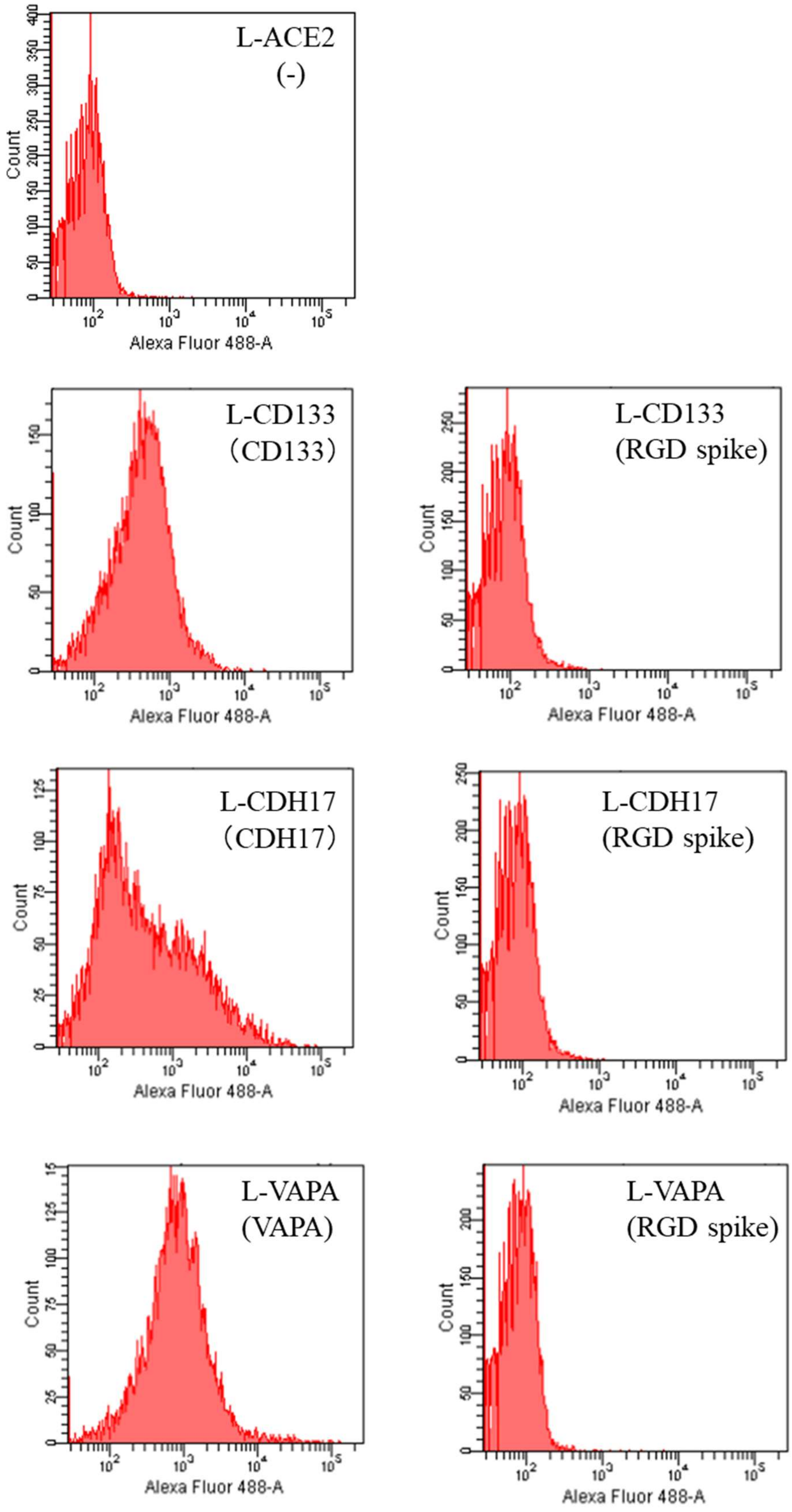
L-ACE2
(-)
L-CD133
（CD133）
L-CD133
(RGD spike)
L-CDH17
（CDH17）
L-CDH17
(RGD spike)
L-VAPA
(VAPA)
L-VAPA
(RGD spike)
Count
Alexa Fluor 488-A

Fig. S2 Characterization of the single candidate molecule-expressing HEK293T cells

Representative data from flow cytometric analysis of L-CD133, L-CDH17, and L-VAPA cells. Firstly, these cells were treated with antibodies against each candidate molecule, followed by incubation with Alexa Fluor 488-conjugated anti-rabbit IgG (L-CD133 (CD133), L-CDH17 (CDH17), and L-VAPA (VAPA); left panel). The cells were also treated with spike protein, followed by Alexa Fluor 488-conjugated anti-mouse IgG (L-CD133 (RGD spike), L-CDH17 (RGD spike), and L-VAPA (RGD spike); right panel). Two independent experiments were performed.

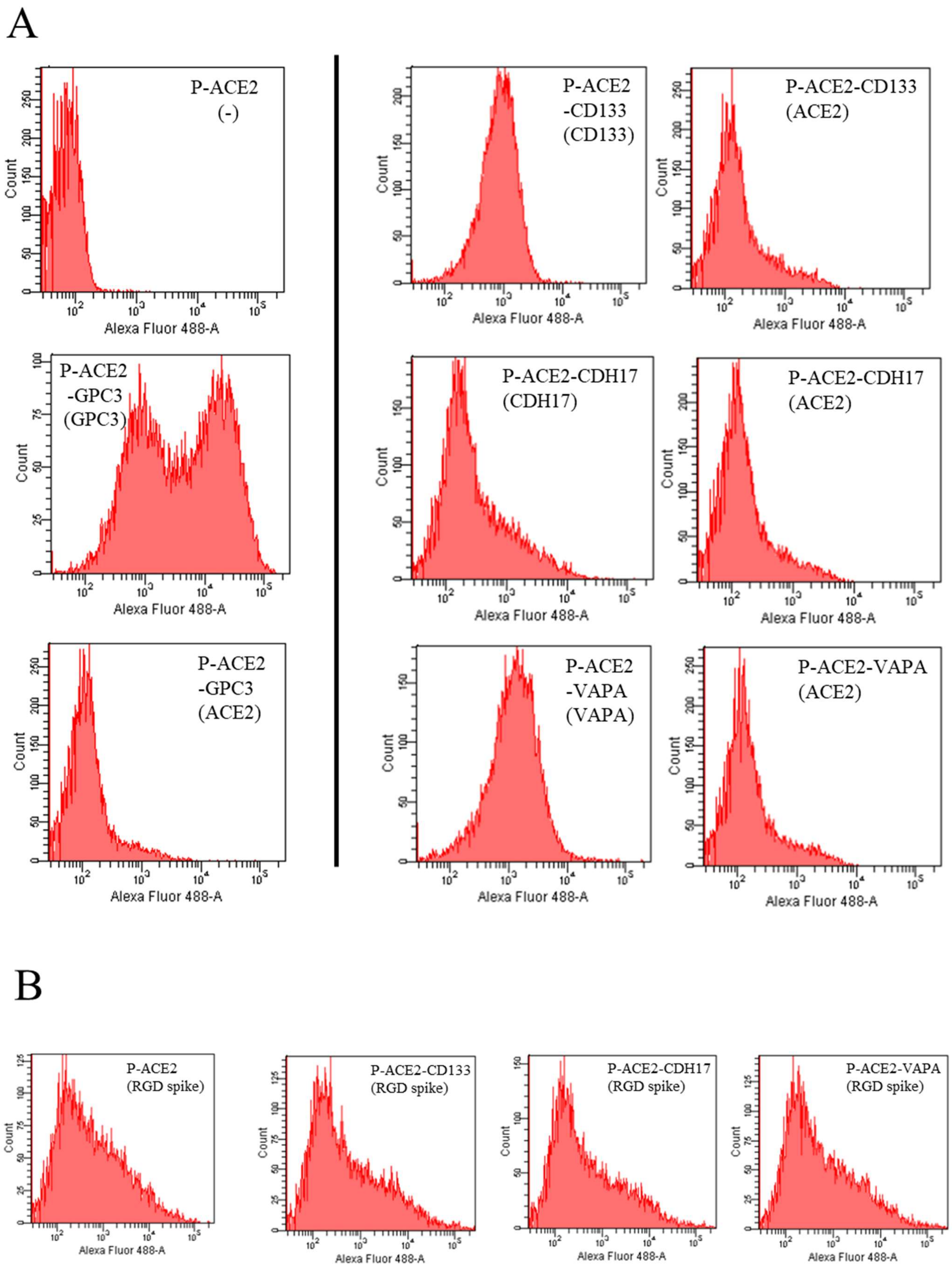
A
P-ACE2
(-)
P-ACE2
-CD133
(CD133)
P-ACE2-CD133
(ACE2)
P-ACE2
-GPC3
(GPC3)
P-ACE2-CDH17
(CDH17)
P-ACE2-CDH17
(ACE2)
P-ACE2
-GPC3
(ACE2)
P-ACE2
-VAPA
(VAPA)
P-ACE2-VAPA
(ACE2)
Count
Alexa Fluor 488-A
B
P-ACE2
(RGD spike)
P-ACE2-CD133
(RGD spike)
P-ACE2-CDH17
(RGD spike)
P-ACE2-VAPA
(RGD spike)

C

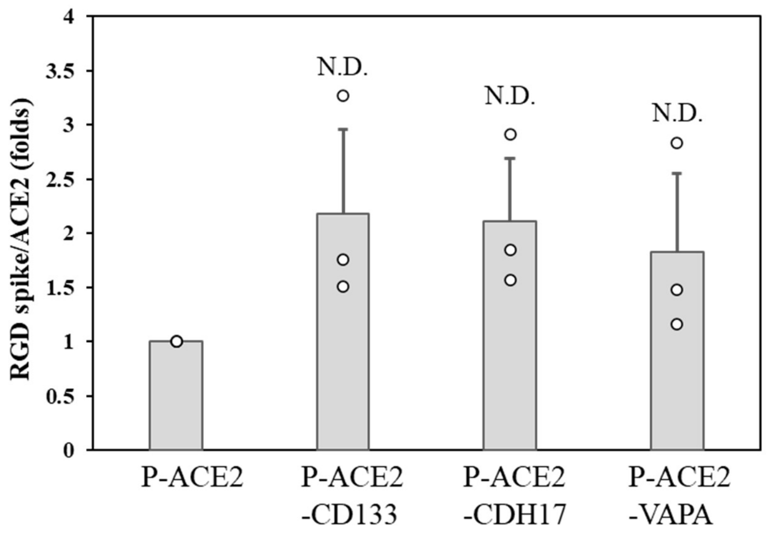

Fig. S3 Characterization of the ACE2- and candidate molecule-coexpressing HEK293T cells

(*A, B*) Representative data from flow cytometric analysis of ACE2- and candidate molecule-coexpressing cells. (*A*) The cells were treated with an anti-ACE2 antibody (P-ACE2-CD133 (ACE2), P-ACE2-CDH17 (ACE2), P-ACE2-VAPA (ACE2), and P-ACE2-GPC3 (ACE2)), first with antibody recognizing each candidate molecule (P-ACE2-CD133 (CD133), P-ACE2-CDH17 (CDH17), P-ACE2-VAPA (VAPA) and P-ACE2-GPC3 (GPC3)), and then with Alexa Fluor 488-conjugated anti-rabbit IgG. Three independent experiments were performed. The data of "P-ACE2 (-)" is the same as the upper right panel of Fig S1A because of the same experimental set. (*B*) The cells were treated with SARS-CoV-2 spike protein (RGD), followed by Alexa Fluor 488-conjugated mouse IgG second antibody (P-ACE2 (RGD spike), P-ACE2-CD133 (RGD spike), P-ACE2-CDH17 (RGD spike), and P-ACE2-VAPA (RGD spike)). Three independent experiments were performed. (*C*) The relative MFI values (RGD spike/ACE2) of P-ACE2-CD133, P-ACE2-CDH17, and P-ACE2-VAPA cells were higher than those of P-ACE2 cells, but no significant difference (N.D.) was observed.

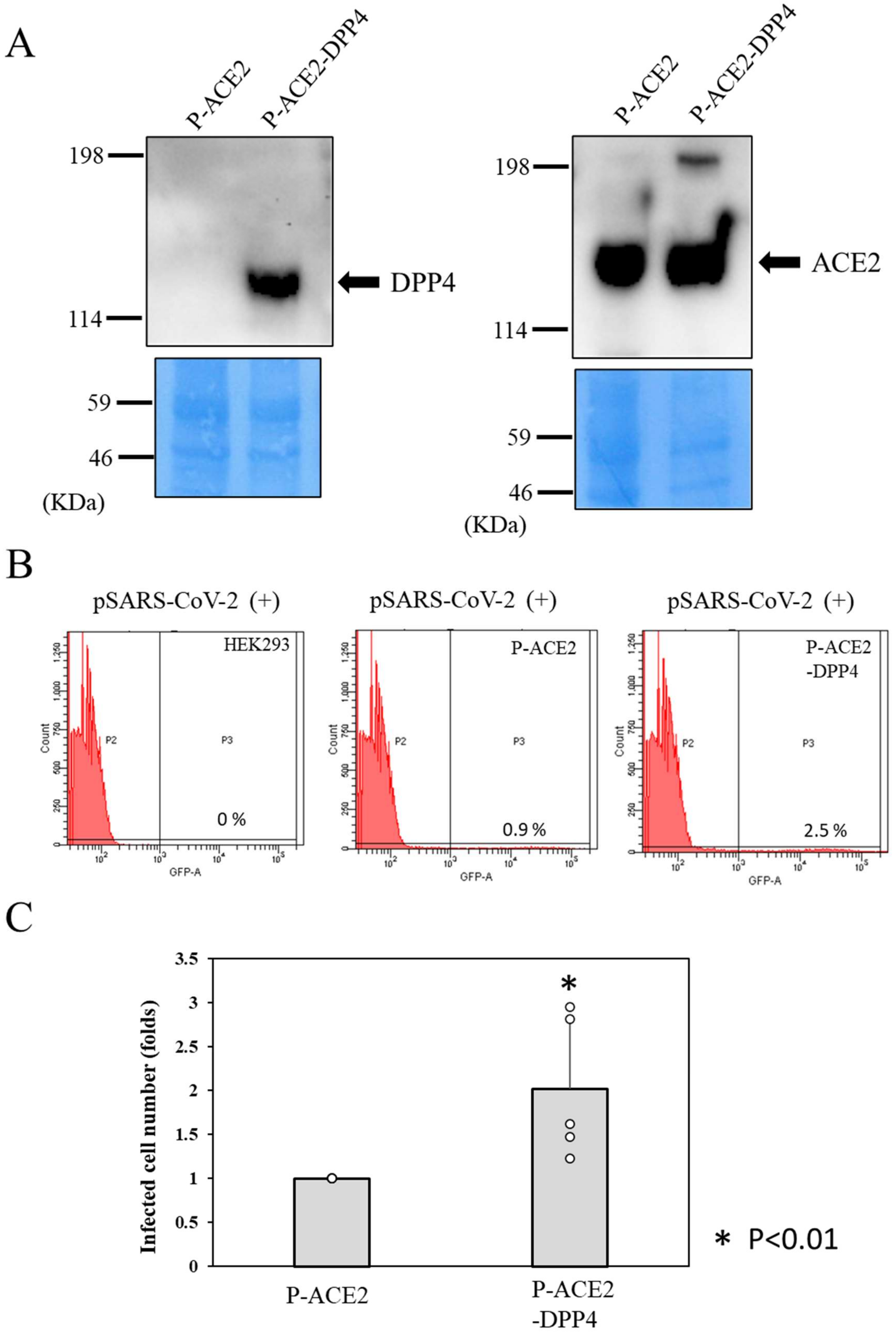
A
P-ACE2
P-ACE2-DPP4
198
114
DPP4
59
46
(KDa)
P-ACE2
P-ACE2-DPP4
198
114
ACE2
59
46
(KDa)
B
pSARS-CoV-2 (+)
HEK293
0 %
pSARS-CoV-2 (+)
P-ACE2
0.9 %
pSARS-CoV-2 (+)
P-ACE2
-DPP4
2.5 %
Count
GFP-A
P2
P3
C
Infected cell number (folds)
3.5
3
2.5
2
1.5
1
0.5
0
P-ACE2
P-ACE2
-DPP4
*
* P<0.01

Fig. S4 *In vitro* infection assay of SARS-CoV-2 pseudovirus for ACE2-DPP4 cells
(*A*) Expression of ACE2 and DPP4 in transfectant HEK293 cells. Western blot analysis of transfectant cell lysates; The cell lysates were subjected to SDS-PAGE (on 8 % gels) and stained with antibodies recognizing ACE2 or DPP4. Arrows indicate bands of the target proteins. The CBB staining image indicates load control. (*B*) Flow cytometric analysis of pSARS-CoV-2-infected cells. Mock HEK293 cells (left panel), P-ACE2 cells (middle panel), and P-ACE2-DPP4 cells (right panel) were analyzed using BD FACS Canto II. GFP-positive cells were defined as the infected cells with a GFP fluorescence intensity of 10^3 or higher (P3 area). Five independent replications were carried out in each experiment. (*C*) Increase in pSARS-CoV-2 infection in P-ACE2-DPP4 cells. The number of GFP-positive cells in each cell was quantified using flow cytometry. The number of infected cells (GFP-positive) in P-ACE2–DPP4 was significantly higher than that in P-ACE2 cells ($P < 0.01$; Wilcoxon rank sum test).

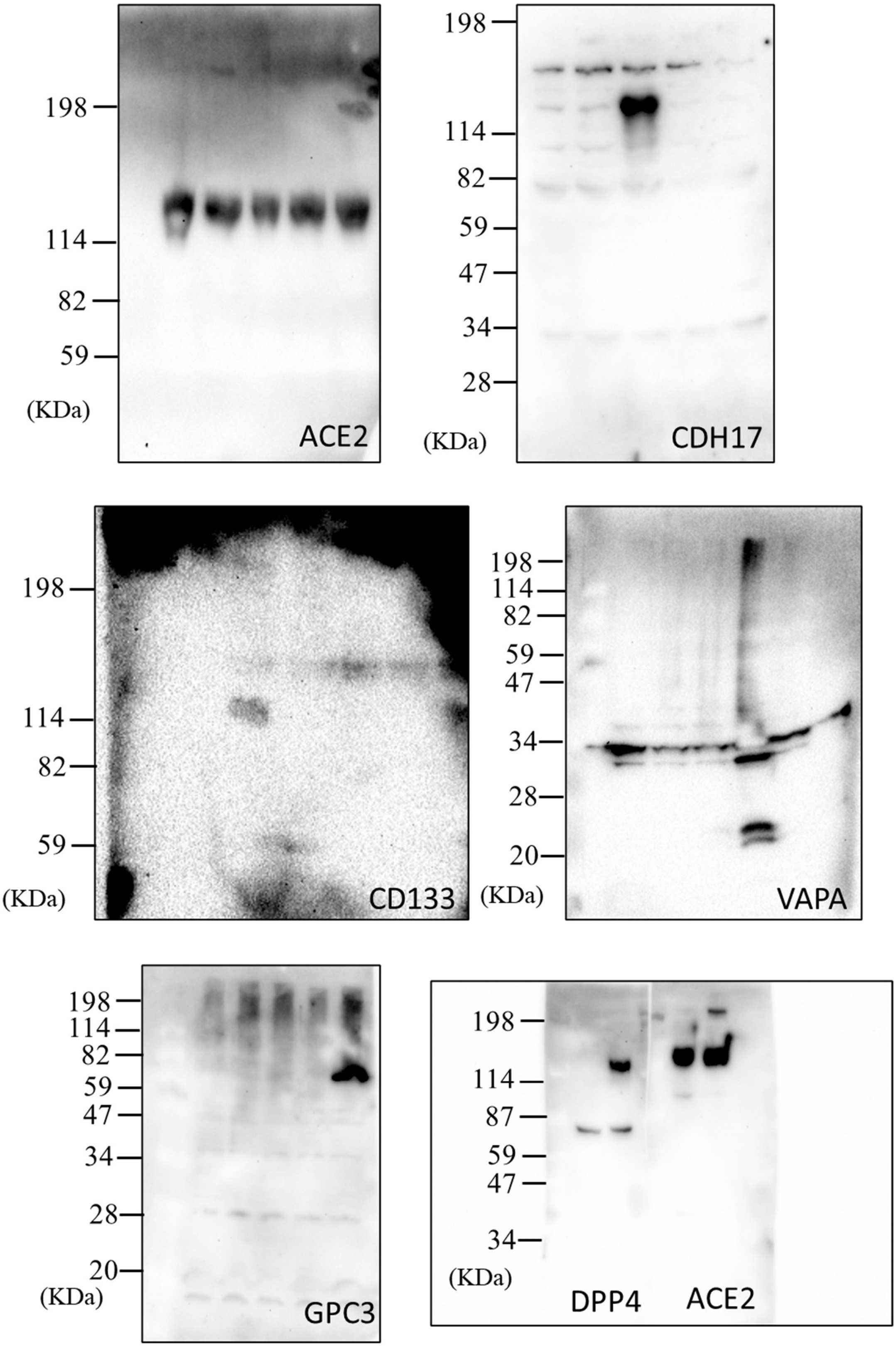

Fig. S5 Entire images of cropped western blot data of Fig. 6A and Supporting Fig. 4
